# An evaluation of semidistributed-pipe-network and distributed-finite-difference models to simulate karst systems

**DOI:** 10.1007/s10040-020-02241-8

**Published:** 2020-11-11

**Authors:** L. W. Gill, P. Schuler, L. Duran, P. Morrissey, P. M. Johnston

**Affiliations:** Department of Civil, Structural and Environmental Engineering, University of Dublin Trinity College, Dublin 2, Ireland

**Keywords:** Karst, Numerical modelling, Groundwater/surface-water relations, Submarine groundwater discharge, Ireland

## Abstract

Several different approaches have been developed to model the specific characteristics of karst aquifers, taking account of their inherent complex spatial and temporal heterogeneities. This paper sets out the development of a semidistributed modelling approach for applications in an Irish karst context using urban drainage software. The models have proven to be very useful for different studies, with examples given for the ecohydrology of ephemeral karst lakes, extreme groundwater-flood alleviation, karst network investigation, submarine groundwater discharge, and quantification of different recharge and flow components. The limitations of the approach are also highlighted, in particular not being able to simulate diffuse infiltration and flow paths explicitly across the groundwater catchment. Hence, a more distributed, finite-difference modelling approach using MODFLOW Unstructured Grid (USG) with the newly developed Connected Linear Network (CLN) process is then compared against the semidistributed approach on the same karst catchment. Whilst it has proven difficult to achieve the same levels of model performance in simulating the spring flows in the distributed model compared to the semidistributed model, the ability to interrogate the flow paths at any point on the three-dimensional aquifer is demonstrated, which can give new insights into flows (and potential contaminant transport) through such complex systems. The influence of the proximity of highly transmissive conduits on the flow dynamics through the much-lower transmissive matrix cells in which the network is embedded has been particularly investigated.

**Electronic supplementary material:**

The online version of this article (10.1007/s10040-020-02241-8) contains supplementary material, which is available to authorized users.

## Résumé

Plusieurs approches différentes ont été développées pour modéliser les caractéristiques spécifiques des aquifères karstiques, prenant en compte leurs hétérogénéités spatiales et temporelles associées à leur complexité intrinsèque. Cet article présente le développement d’une approche de modélisation semi-distribuée appliquée à un contexte karstique irlandais en utilisant un logiciel de drainage urbain. Ces modèles se sont avérés très utiles pour différentes études, avec des exemples appliqués à l’écohydrologie de lacs karstiques éphémères, l’atténuation de crues extrêmes liées aux eaux souterraines, à l’étude du réseau karstique, à la décharge des sources sous-marines et à la quantification des différentes composantes aussi bien de la recharge que du débit. Les limites de cette approche sont aussi mises en évidence, avec en particulier l’incapacité de simuler l’infiltration diffuse et les voies d’écoulement de manière explicite au sein du bassin d’alimentation d’eaux souterraines. Par conséquent, une approche de modélisation plus distribuée aux différences finies utilisant le logiciel MODFLOW avec une grille non structurée (USG) intégrant le processus récemment développé de réseau linéaire connecté (CLN) est ensuite comparée à l’approche semi-distribuée appliquée au même bassin karstique. Alors qu’il s’est avéré difficile d’atteindre les mêmes niveaux de performance du modèle concernant la simulation du débit à la source à l’aide du modèle distribué en comparaison au modèle semi-distribué, la possibilité d’examiner les voies d’écoulement en tout point de l’aquifère en 3D est démontrée, ce qui peut donner de nouvelles connaissances sur les écoulements (et le transport potentiel de contaminants) au sein de tels systèmes complexes. L’influence de la proximité de conduits hautement transmissifs sur les écoulements dynamiques au travers des cellules de transmissivité plus faible de la matrice au sein de laquelle le réseau est intégré, a été particulièrement étudiée.

## Resumen

Se han elaborado varios enfoques diferentes para modelar las características específicas de los acuíferos kársticos, teniendo en cuenta sus complejas y propias heterogeneidades espaciales y temporales. En el presente documento se expone la elaboración de un enfoque de modelización semidistribuido para aplicaciones en un contexto kárstico de Irlanda utilizando programas informáticos de drenaje urbano. Los modelos han demostrado ser muy útiles para diferentes estudios, y se dan ejemplos para la ecohidrología de los lagos kársticos efímeros, la mitigación de las inundaciones extremas de aguas subterráneas, la investigación de redes kársticas, la descarga submarina de aguas subterráneas y la cuantificación de diferentes componentes de la recarga y el flujo. También se ponen de relieve las limitaciones del enfoque, en particular el hecho de no poder simular explícitamente la infiltración difusa y las trayectorias de flujo a través de la cuenca de captación de aguas subterráneas. Por lo tanto, un enfoque de modelización de diferencias finitas distribuidas utilizando la red no estructurada (USG) de MODFLOW con el proceso de la Red Lineal Conectada (CLN) recientemente desarrollado se compara entonces con el enfoque semidistribuido en la misma cuenca kárstica. Si bien se ha demostrado que es difícil lograr los mismos niveles de rendimiento del modelo en la simulación de los flujos de manantiales en el modelo distribuido en comparación con el modelo semidistribuido, se demuestra la capacidad de interrogar las trayectorias de los flujos en cualquier punto del acuífero tridimensional, lo que puede dar nuevos conocimientos sobre los flujos (y el transporte de contaminantes potenciales) a través de sistemas tan complejos. Se ha investigado en particular la influencia de la proximidad de conductos altamente transmisivos en la dinámica de los flujos a través de las células de la matriz transmisiva, mucho más bajas, en las que está incorporada la red.

## 摘要

鉴于岩溶含水层内在的复杂时空异质性, 已经开发出几种不同的方法来模拟岩溶含水层的特定性质。本文提出了基于城市排水软件的半分布式模型的开发方法, 并在爱尔兰喀斯特环境中应用。该模型已被证明对不同的研究非常有用, 并举例说明了季节性的喀斯特湖的生态水文学, 极端地下水洪涝减缓, 岩溶网络调查, 海底地下水排泄以及不同补给量和流动量的量化。还强调了该方法的局限性, 特别是不能明确模拟整个地下水盆地的扩散入渗和流动路径。因此, 然后将MODFLOW非结构化网格(USG)和新开发的连接线性网络(CLN)过程耦合的分布式有限差分方法与同一个喀斯特流域的半分布式方法进行了比较。尽管已证明分 布式模型模拟的泉流量难以达到与半分布式模型相比相同水平的模型性能, 但仍展示了在三维含水层上任意点处查询流路的能力, 这可以给出分析该类复杂系统流量(以及潜在的污染物传输)的新方法。特别研究了高传输管流的邻近度对通过嵌入网络的传导率低得多单元的流动动力学的影响。

## Resumo

Diversas abordagens diferentes foram desenvolvidas para modelar as características específicas dos aquíferos cársticos, levando em consideração suas complexas heterogeneidades espaciais e temporais inerentes. Este artigo apresenta o desenvolvimento de uma abordagem de modelagem semidistribuída para aplicações em um contexto cárstico irlandês usando software de drenagem urbana. Os modelos provaram ser muito úteis para diferentes estudos, com exemplos dados para a ecohidrologia de lagos cársticos efêmeros, alívio de inundações subterrâneas extremas, investigação de rede cárstica, descarga de água subterrânea submarina e quantificação de diferentes componentes de recarga e fluxo. As limitações da abordagem também são destacadas, em particular não ser capaz de simular infiltração difusa e caminhos de fluxo explicitamente através da captação de água subterrânea. Portanto, uma abordagem de modelagem de diferenças finitas, mais distribuída, usando MODFLOW Unstructured Grid (USG) com o processo de Rede Linear Conectada (RLC) recém-desenvolvido é então comparada com a abordagem semidistribuída na mesma bacia cárstica. Embora tenha se mostrado difícil alcançar os mesmos níveis de desempenho do modelo na simulação dos fluxos de nascente no modelo distribuído em comparação com o modelo semidistribuído, a capacidade de interrogar os caminhos de fluxo em qualquer ponto do aquífero tridimensional é demonstrada, o que pode dar novas percepções sobre os fluxos (e o transporte potencial de contaminantes) por meio de tais sistemas complexos. A influência da proximidade de condutos altamente transmissivos na dinâmica do fluxo através das células da matriz transmissiva muito inferior nas quais a rede está inserida foi investigada particularmente.

## Introduction

Karst aquifers host some of the most important drinking water resources across the world with roughly one quarter of the global population estimated to be relying on the supply of freshwater from these aquifer types (Ford and Williams 2007). In Europe, approximately 22% of the land surface of the continent is underlain by (more or less) karstified carbonate rocks (Goldscheider et al. 2020). Karst systems also support many unique ecological habitats such as calcareous fens, ephemeral lakes (poljes and turloughs), cenotes etc., many of which provide habitat for floral and faunal species of international importance (Irvine et al. 2018). In Europe, for example, these wetlands are designated as groundwater dependent terrestrial ecosystems (GWDTEs) under the EU Water Framework Directive (WFD) (2000/60/EC) and many are listed as a priority habitat on Annex I of the EU Habitats Directive (EEC/92/43).

Karst systems are complex systems to unravel, being highly heterogeneous geological formations characterised by multi-scale temporal and spatial hydrological behaviour. They contain a mix of primary (matrix), secondary (tectonic) and tertiary (dissolution) porosity with several orders of magnitude between the associated permeabilities (White and White 2005). Fractures and bedding planes provide the framework for the karstification processes during which ongoing dissolution develops a conduit network. Such aquifers are often characterized by a hierarchical drainage system enveloped in the much lower hydraulic conductivity matrix. Whilst the apertures through which water can move are a continuum ranging between voids (>10 μm) up to accessible conduits or caves (depending on the level of karstification), permeabilities are generally categorised in terms of two or three flow and recharge components (Atkinson 1977; White and White 2005). Hence, depending on the evolutional state of a karst aquifer, diffusely recharged groundwater may travel through different sized openings, from small fissures towards conduits, being subject to different flow dynamics en route ranging between laminar and fully turbulent flow (Kovács et al. 2005; Giese et al. 2018). Infiltration processes vary from diffuse, slow infiltration through small matrix and fracture volumes, to concentrated, rapid infiltration directly into the channel network (via swallow holes/dolines for example). Equally, discharge conditions differ, from diffuse seepage to concentrated discharge at karst springs from the conduit network.

The earliest attempts to model karst aquifers explicitly in terms of their unique hydrogeological processes seemed to be in the mid-1970s (e.g. Thrailkill 1974; Kiraly 1975; Mangin 1975). Since then, many different approaches have evolved from global (lumped parameter) models through to more physically based distributed models, with various permutations/forms of semidistributed models in between (Kovács and Sauter 2007; Ghasemizadeh et al. 2012).

Given the inherent heterogeneity of karst systems, different deterministic modelling approaches have been formulated using a global response to relate an input rainfall signal to an output spring discharge using a transfer function (e.g. Dreiss 1982). These so-called black box models contain no spatial information or explicit representation of any physical processes on the karst aquifer. Such models can be formulated using transfer functions, i.e. a form of ‘instantaneous unit hydrograph’ determined from a conceptual model (for example the Nash-reservoir model); the parameters are then optimised to fit the data (Denić-Jukić and Jukić 2003; Fleury et al. 2007; Jukić and Denić-Jukić 2008). A commonly used tool called KarstMod provides an adjustable modular structure of several reservoirs in parallel/series as a modelling platform for karst systems (Mazzilli et al. 2017). Another approach, the VarKarst model, defined as a semi-lumped model (i.e. more of a grey box model), considers the spatial variability of the soil and epikarst characteristics by distribution functions (Hartmann et al. 2012, 2013; Mudarra et al. 2019). Due to the time-invariant limitations of some transfer function techniques when trying to reconstruct the temporal structure of relatively infrequent flow events such as floods or low water periods (Kuczera and Mroczkowski 1998; Labat et al. 2000a), different numerical methods have been used to develop nonparametric transfer functions in order to capture nonlinear and nonstationary dynamics more accurately. These have included approaches more rooted in the spectral (frequency) domain (Larocque et al. 1998; Labat et al. 2000a, 2002; Bailly-Comte et al. 2008) as well as time/frequency domains using wavelets (Labat et al. 2000b, 2001; Schuler et al. 2020b). Machine learning approaches, for example artificial neural networks, have also been applied to karstic aquifers (Beaudeau et al. 2001; Kurtulus and Razack 2007; Hu et al. 2008*).*

Then there are the models that attempt to simulate the internal geometries and associated hydraulic response of karst aquifers, in particular the conduit domain modelled as pipe networks based upon hydraulic engineering concepts and calculations (open channel flow, pressurised flow etc.). Often these more hybrid-type, semidistributed models use conceptual reservoirs to control areal recharge processes which lack spatial resolution, but then specific pipe network morphometry is used to control specific functions such as springs at different elevations (Halihan et al. 1998) or a syphon in the case of rhythmic springs (Bonacci and Bojanić 1991). Several recent models have employed urban drainage software to model the hydraulic dynamics of conduit networks accurately (including ponors, estavelles, etc.), particularly using SWMM (Chen and Goldscheider 2014), including incorporating groundwater/surface-water interactions in poljes (Mayaud et al. 2019) and turloughs (Gill et al. 2013a; see section ‘Semidistributed pipe network models (development in Ireland)’).

More physically distributed modelling approaches divide the karst aquifer up in two- or three-dimensional (2D or 3D) grids, usually quite small in scale. These require much higher levels of input information, with hydraulic parameters and system states defined for each grid cell (Hartmann et al. 2014). Some models have been developed upon the conceptual approach that the hydraulic heterogeneities throughout the whole aquifer can be averaged out and represented by a fully equivalent porous medium (Scanlon et al. 2003). However, such an approach will not allow discrete concentrated fast flows to be simulated explicitly and can lead to significant underestimates of flow-through or residence times, especially in smaller-scale aquifers. This could lead to misconceptions over contaminant transport, with the notable *E. coli* infection incident at Walkerton in Canada being an infamous example (Worthington and Smart 2017). Other modelling approaches characterise the physical attributes of karst aquifers more explicitly, allowing both the high transmissivities of conduits and low transmissivity of the matrix to be simulated at the same time. This includes the double continuum approach where the matrix and the karst conduits are modelled as two interacting continua (Teutsch and Sauter 1998), and the combined discrete-continuum approach in which the karst conduit network is embedded as discrete elements inside the matrix. This has been applied in many theoretical studies of karst processes (Reimann et al. 2011) and of karst evolution (Liedl et al. 2003; Borghi et al. 2012). This approach is also now possible in the widely used groundwater model MODFLOW which has incorporated the Conduit Flow Process (CFP) module into its code in order to simulate turbulent flow, although few studies have been published to date using this for karst systems (Chang et al. 2019). A more recent development is MODFLOW Unstructured Grid (USG), applied with the newly developed Connected Linear Network (CLN) process for karst aquifers, as presented in section ‘Materials and methods’. A major challenge for potential application of physically distributed karst models is that the location and the size of conduits are generally unknown in most aquifers (Borghi et al. 2016), making such data-demanding models questionable against the more general debate about the over-parameterisation of hydrologic models (Beven 2006).

Overall, the choice of modelling approach taken usually depends on the main purpose for which it is being developed—to simulate spring flow, to evaluate the ecohydrology of wetlands, to simulate and predict groundwater-surface water interaction (i.e. flooding), to evaluate water contaminant transport and attenuation processes for groundwater protection etc. This paper sets out the development of semidistributed-pipe-flow models for applications in an Irish karst context using urban drainage software applied in an Irish context over the past 15 years, with a critical review of the strengths and weaknesses, and then goes on to compare such a semidistributed pipe network model against a new approach using a more distributed, finite difference model, MODFLOW USG with CLN on the same catchment.

## Semidistributed pipe network models (development in Ireland)

This section summarises the historical evolution of Irish pipe network models in the specific context of Irish karst hydrogeology and main research questions answered.

### Overview of Irish karst

Limestone forms the bedrock of more than 40% of the Republic of Ireland with most karst being lowland, situated less than 100 m above current sea level, in comparison to a few more “typical” upland/plateau karst areas located at their margins (Drew 2018). By far, the most widespread carbonate rock is from the Carboniferous, which has experienced many episodes of karstification since the beginning of the Dinantian, most significantly the most recent Cenozoic Era (Drew 2018). Ireland experienced relatively fast climate oscillations between glacial and warmer interglacial periods during the Quaternary (Edwards and Craven 2017), with the main karstification—the development of active karst conduit flow systems and a weathered epikarst zone–occurring during the warmer phases. The relative sea-level has also fluctuated as a result of these successive glaciation events, thus changing the hydraulic gradients through karst systems; for example, the sea-level was estimated to be 60–100 m below present levels in Galway Bay since the last glaciation event around 26,000 years ago (Edwards and Craven 2017; O’Connell and Molloy 2017).

Much of the Irish karst is buried under a thick layer of glacial deposit with no dramatic karst features on show at the surface. The overburden conceptually dampens diffuse autogenic recharge into the underlying bedrock. Where low-lying, karst may exhibit groundwater/surface-water interactions following periods of intense rainfall where the underlying karst network cannot take the hydraulic load and water surcharges to the surface to form temporary lakes known as turloughs. The dynamics of these lakes, both from an ecohydrological perspective but also due to more damaging extreme flood events, became the focus of the development and evolution of semidistributed pipe network models in Ireland, as follows.

### 1D models to simulate groundwater/surface-water interaction

**Fig. 1:**
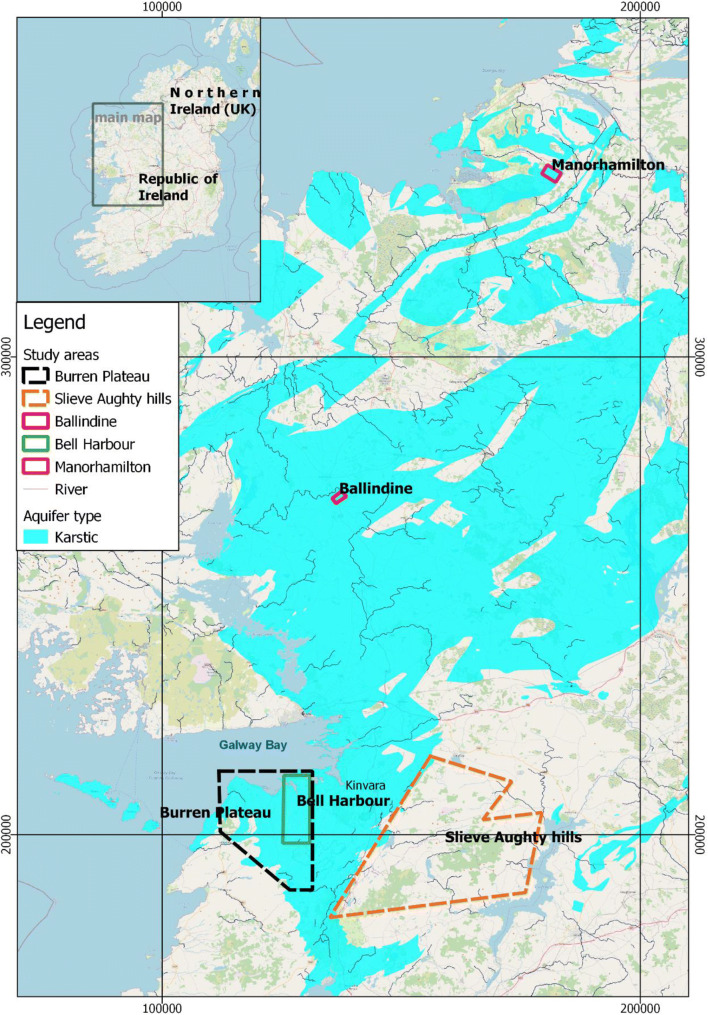
Map of Ireland showing locations of the main sites referred to in these studies

**Fig. 2:**
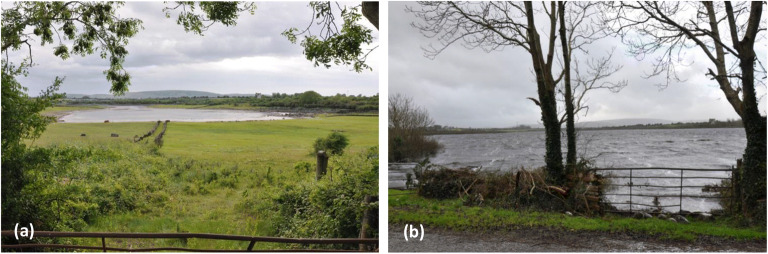
Caherglassaun turlough: **a** almost empty in summer and **b** flooded in winter

The first pipe network models were focussed on the lowland karst area of South Galway in the west of Ireland which has experienced extensive flooding during six winters over the past 20 years (1989/1990, 1991, 1994, 1995, 2009 and 2015/2016). The area, a catchment of approximately 500 km^2^ receives allogenic runoff from about one third of the catchment area from three main rivers draining the Old Red Sandstone Slieve Aughty Hills—see Fig. 1 and Fig. S1 in the electronic supplementary material (ESM). The lowland karst aquifer is fed by these sinking streams which disappear into underground fissures and conduits and then frequently reappear again in surface reaches or as turloughs in glacially formed depressions. The drainage takes the flows underground to the north-west to the Atlantic Ocean at Kinvara through a complex multi-level conduit system in this lowland network that has formed as a result of past glaciation cycles and their impact of karstification processes, as discussed previously (Naughton et al. 2018). The turloughs flood in winter and store temporarily huge volumes of water, forming a key component in the hydrogeological regime. They behave as surge tanks in the system, attenuating flow in the subterranean conduit network. In summer, however, these turloughs normally dry out (see Fig. 2).

The serious flooding in the mid-1990s stimulated the largest flood study in Ireland to address groundwater flooding problems (Southern Water Global 1998) which led to a model using urban drainage software *Hydroworks* (Wallingford software) being developed. Unlike many karst systems there were no discharge hydrograph data available (the outlet spring for the system being intertidal), with insights into the system gained from the analysis of turlough flooding time series over the area, as well as some caving records, tracing studies and borehole monitoring.

Over the next 10 years, water level data continued to be collected in the main turloughs (Blackrock, Coy, Coole, Garryland and Caherglassaun) in parallel to new integrated multi-disciplinary research interest in the ecohydrology of the ephemeral wetlands (Naughton et al. 2012; Porst et al. 2012; Waldren et al. 2015). The allogenic flows into the lowland karst network from the three main rivers draining off the Slieve Aughty Hills were constantly gauged at locations on the edge of the sandstone before disappearing into the karst limestone, as well as the tidal levels in Galway Bay into which the system drains, i.e. the downstream boundary condition. Rain gauges were positioned across the catchment to assess the spatial distribution of rainfall. Turloughs were accurately surveyed during the summer periods when the waters had receded using a differential GPS system, from which contour maps were plotted and depth-area-volume relationships derived.

A new semidistributed 1D model of the catchment was developed to simulate groundwater-surface water interaction for turloughs (Gill et al. 2013a). This was based on the drainage software *InfoWorks CS* version 8.5 (Wallingford Software) due to its ability to model the hydraulics of the karst conduit network in both open channel and pressurised pipe flow. The governing model equations are the Saint-Venant equations of conservation of mass and momentum. For pressurized pipe flow the model incorporates the conceptual vertical and narrow Preissmann slot into the pipe soffit to provide a free surface condition for the flow when the water level is above the top of a closed conduit, enabling a smooth transition between the free surface and surcharged conditions. The groundwater/surface-water turlough dynamics were modelled as storage ponds in the software which were configured with the same depth-volume characteristics as the surface topography, as derived from the detailed field surveying.

**Fig. 3:**
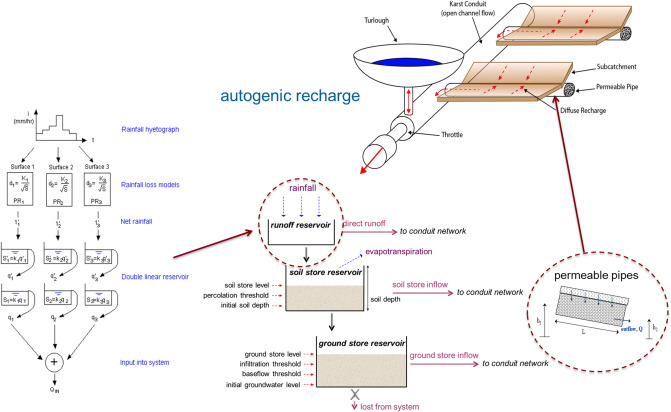
Autogenic rainfall recharge (adapted from Gill et al. 2013a and McCormack et al. 2017)

Diffuse recharge from rainfall is modelled per subcatchment via a series of reservoirs: rainfall runoff, soil and groundwater stores in series, all yielding different delayed discharges in parallel into the pipe network (see Fig. 3). All flows discharge into permeable pipes, one connected for each subcatchment to represent the primary and secondary permeability expressed by Darcy’s Law. The allogenic recharge into the karst network from the rivers was input as point flow time series into the head of the pipe network. These data were derived by three separate rainfall-runoff models one for each river, using the MIKE11-NAM software (DHI Software).

**Fig. 4:**
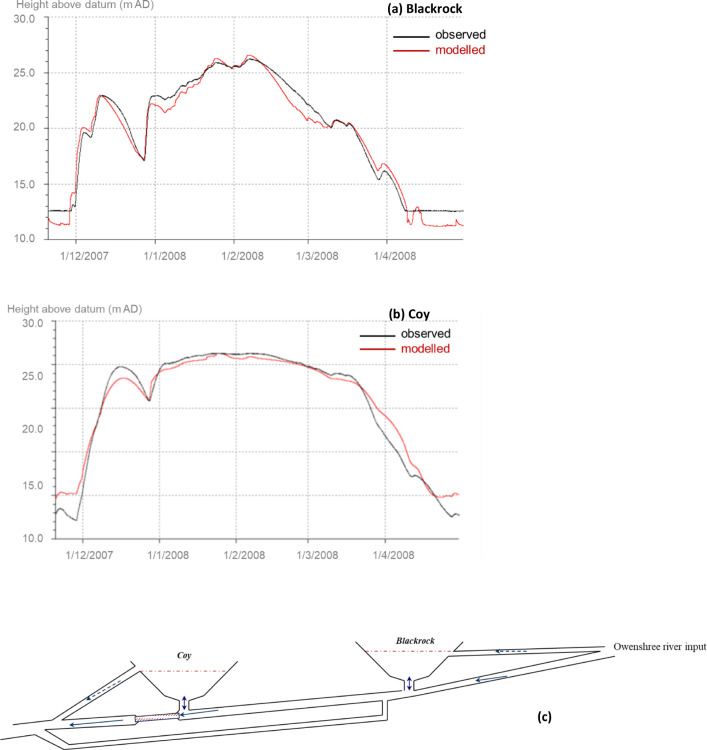
**a** Blackrock and **b** Coy turlough simulation results (black is observed data, red is simulated data) and **c** the final modelled hydraulic network configuration (Gill et al. 2013a)

The network was developed on the back of previous field investigations (tracer studies, caving records etc.), and further insights into the system were gained from the accumulating hydro-meteorological data across the catchment as well as water hydrochemistry (Gill et al. 2018). Time and frequency series analyses on the continuous water level measurements of the five turloughs in the linked network were used in order to elucidate the nature of the hydraulic pipe configurations at key points in order to improve the conceptual model (Gill et al. 2013b). Good correlations for multiple years were achieved across the five turlough water-level fluctuations on the network, achieving Nash-Sutcliffe efficiencies (NSE) ranging from 0.85 to 0.97 (see Fig. 4).

This model was improved in following years and used to compare different approaches to attempt to define the submarine groundwater discharge into Kinvara Bay (McCormack et al. 2014), as well to gain insights into the nutrient processes in the turloughs and wider catchment using non-reactive solute tracer against field data (McCormack et al. 2016). The main limitation of this semidistributed modelling approach based on urban drainage models that had become apparent, however, was the lack of spatial resolution possible with regard to the diffuse recharge component, as it is homogenised on a subcatchment basis and then simplified by the series of conceptual reservoirs. There was very little detailed recharge assessment carried out in the field against which to calibrate such diffuse flow contributions, but nevertheless, whilst not a direct problem for these applications, it does limit the approach in terms of further use of the model to simulate contaminant transport in any detail.

### 1D models for more investigative purposes

The 1D modelling approach was then extended towards a neighbouring area, namely Bell Harbour, a coastal-upland system of the Burren karst plateau (see Fig. S2 of the ESM). Much less information was available about this catchment compared to the lowland karst area (see section ‘1D models to simulate groundwater/surface-water interaction’) adjacent to it. It was presumed that there must be drainage off the Burren northwards to the sea following the steep topography, but there was no clear evidence of a significant spring in the Bell Harbour Bay. In the valley there is one turlough and one borehole which were monitored using level loggers for a period of 2 years. Some previous geophysical work (McCormack et al. 2017) suggested the presence of a major shallow conduit extending south–north following the topography of the valley. Monitoring was subsequently extended and intensified, including the collection of high-resolution climate data and salinity measurement at the outlet of Bell Harbour. The latter were used to estimate the submarine and intertidal groundwater discharge (SiGD) into the bay. From this a water balance was derived which indicated that the SiGD into the bay was much lower than what would be expected especially during low recharge (summer) periods. Single borehole dilution tests confirmed deep groundwater flow <–170 m below sea level, suggesting that the catchment is largely drained via deep conduits bypassing Bell Harbour Bay, explaining the unmatched water balance (Schuler et al. 2018). Hence, this deep discharge component would occur further out into the sea via submarine springs. The presence of such deep conduits must be related to past sea-level fluctuations, presumably beyond the last glacial maxima.

**Fig. 5:**
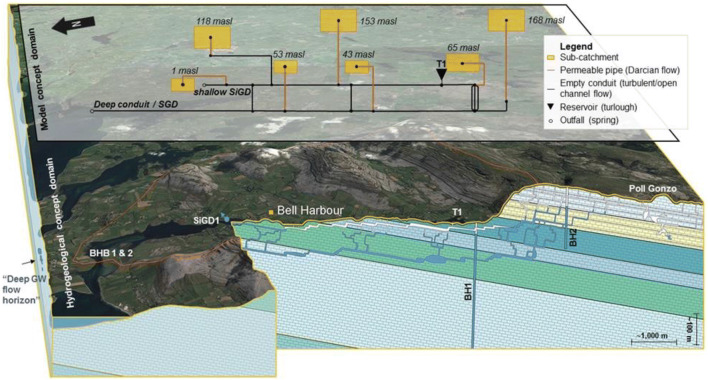
3D conceptual model of the hydrogeology at the Bell Harbour catchment, including conduit networks that are resembled in the pipe network model. Yellow boxes represent the conceptual sub-catchments with mean elevations (Schuler et al. 2018)

The Bell Harbour system was therefore conceptualised as a multi-level-conduit-dominated coastal karst aquifer, governed by elevation (head) differences transmitted through conduits, governing the seasonality of discharge and turlough flooding. The plausibility of this functioning was numerically confirmed by a pipe network model (Fig. 5; Schuler et al. 2018). A tracer test was subsequently carried out between the upland of the Bell Harbour catchment (Burren Plateau) and the shoreline and sea, confirming (deep) groundwater flow and submarine groundwater discharge into the Atlantic Ocean (Schuler et al. 2020a).

The model performed relatively well in simulating the timing of estimated discharge, yet, the magnitude per event was often exaggerated. Another major drawback of this modelling approach which was identified is the missing ‘storage’ connected to the conduits: when the pipes surcharge, water cannot be pushed into a subsystem of primary or secondary porosity. Instead, it remains in the pipe until being discharged. This aspect relates to the functioning of the soil and groundwater store (Fig. 3) that can only discharge (but not receive backwards flow). Hence, a storage component is missing, presumably resulting in exaggerated discharge events.

### 1D/2D models to determine flood alleviation possibilities

Following more damaging flooding in the south Galway catchment in 2009 and 2015/2016, renewed interest was stimulated to provide an engineered flood alleviation solution for the area (Naughton et al. 2017). Many more locations across the catchment were instrumented (additional turloughs, rivers etc.) and more detailed topography across the entire area was derived from LiDAR high-resolution digital terrain model at 1 m grid resolution.

The original 1D model (see section ‘1D models to simulate groundwater/surface-water interaction’) was significantly developed on *InfoWorks ICM* (Innovyse; Morrissey et al. 2020) and certain key floodplains were also included as 2D areas in the model. In order to gain efficiencies in model computation, terrain-sensitive meshing was utilised whereby the resolution of the mesh is increased in areas where large variations in height occur. The maximum height variation allowed during the generation of the mesh was 0.25 m. The 1D pipe network (representing flow within the karst bedrock) was linked to this 2D mesh using either 2D nodes or inline banks. Flow between the 2D mesh towards a 1D node (and vice versa) was based on standard head-discharge relationships. Flow between the 1D node and the 2D mesh was based on the weir equation, the benefit of using an inline bank over a 2D node being that it allowed the transfer of flow between 1D and 2D, and vice versa, along a number of mesh elements as opposed to at one discrete point. In this regard, inline banks were utilised where basins spilled into overland channels with 2D nodes used to represent swallow holes in the karst. The major constraint in this approach was the computing power required to perform calculations for the 2D mesh elements over such large areas with simulations taking days to complete which made calibration an onerous task. In addition, representing the flow over the weirs between the 1D/2D interface was difficult to calibrate due to the lack of real data to base the calibration against.

**Fig. 6:**
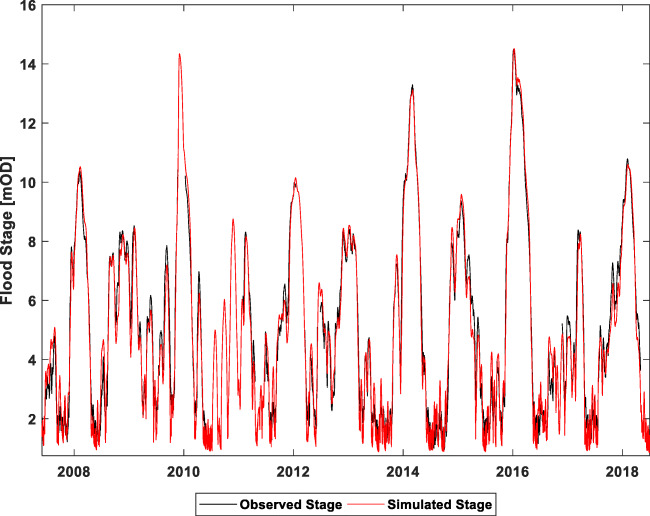
Long-term times series of modelled levels of Caherglassaun turlough showing simulation of the two extreme flood events in 2009 and 2015/2016

The model was calibrated over much longer time series of turlough water-level data (>10 years) with a particular focus on matching the peak flood events in 2009 and 2015/2016 (Fig. 6). Extremely high calibrations against water levels and associated volumes were obtained with average NSE and Kling-Gupta efficiencies (KGE) across 12 flood locations of 0.89 and 0.85 respectively.

**Fig. 7:**
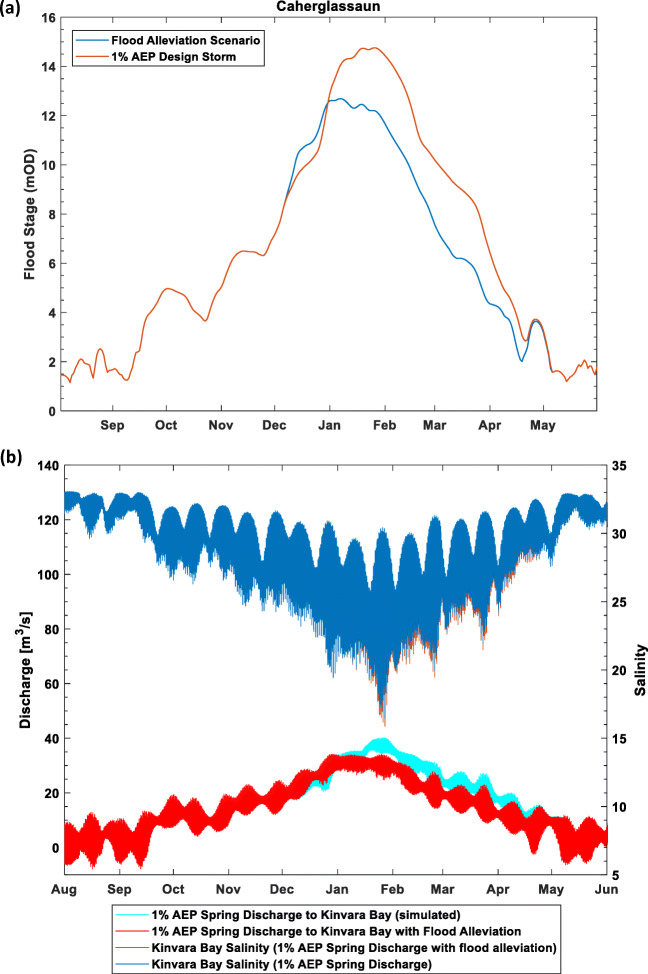
Impact of 1% AEP (annual exceedance probability) flood event on **a** water levels in Caherglassaun turlough (with and without flood alleviation overflow channels) and **b** salinity in the Kinvara Bay from the simulated freshwater discharge

The model has allowed the impact of potential flood alleviation channels at strategic locations in the catchment to be quantified with respect to reduction in peak flood levels in the turloughs against a range of different rainfall scenarios, including climate change predictions. The model has also been used to assess potential impacts in Kinvara Bay, where the spring discharges, in terms of additional reductions in salinity due to freshwater being diverted overland during large flood events (Fig. 7). Such higher freshwater flows might impact on commercial shell fishing activities in the bay. These predicted freshwater flows are being used as inputs to more detailed hydro-dynamic modelling of the bay.

Whilst this version of the model was significantly expanded and was calibrated over a long-term period, the diffuse recharge process (through the soil and epikarst) still lacked data with which to calibrate the subcatchment reservoirs and permeable pipes. This was not deemed to be a major issue for peak flood events which was the focus of this work; however, the filling and recessions of the flood hydrographs for less extreme events could be more accurately represented with a better understanding of the epikarst storage and flow components. In addition, whilst stable and accurate 2D models were achieved, there were challenges in connecting the 1D/2D interface in a manner which was representative and it was difficult to achieve very accurate results which could be used for design purposes for example. Water was slower wetting up the 2D mesh and delayed in draining due to poor calibration either at the 2D nodes or inline banks which were difficult to refine in a representative manner. The 2D simulation computing power required was another significant limitation of the model and severely hampered the calibration process.

### Characterise the diffuse component by use of numerical techniques

**Fig. 8:**
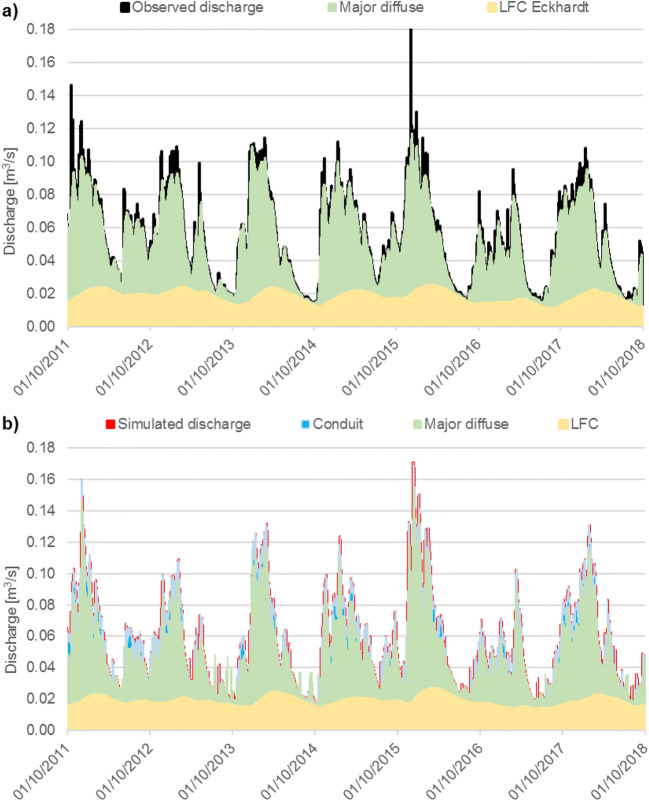
Summary of recharge and flow components at Ballindine spring for **a** observed and **b** simulated time series for the hydrological years 2012 to 2018 (Schuler 2020)

Given the limitations highlighted in the previous models about the uncertainty in the diffuse recharge process, two catchments with long-term, high-resolution spring discharge time series were chosen, namely Ballindine and Manorhamilton (see section ‘Materials and methods’) in order to characterise the different flow components better by use of numerical techniques in both time and frequency domains on output signals (Schuler et al. 2020b). The quantification of the relative contribution of these components, as well as their numerical representation, was attempted by analysing the three recharge components in the time and frequency domain. While the analysis in the time domain follows traditional approaches, the analysis of the power spectrum allows frequencies associated with specific spectral coefficients and noise types to be distinguished more objectively. The analysis is based on the hypothesis that the different frequency-noise components are the result of aquifer heterogeneity transforming the random rainfall input into a sequence of non-Gaussian signals (Labat et al. 2000b; Massei et al. 2006; Duran et al. 2020). The distinct signals were then numerically represented in the context of semidistributed pipe network models (*InfoWorks*) to simulate the recharge, flow and discharge of these two karst systems more realistically, as shown in Fig. 8 for the Ballindine spring (see Fig. S3 of the ESM for catchment), which is largely influenced by river-bed exfiltration (Schuler et al. 2020a). This approach exemplifies the conjunctive use of frequency and time domain analysis in conceptualisation of a hydrological system along with modelling and evaluation.

## Materials and methods

### Study area

#### Geography, geology, structure

**Fig. 9:**
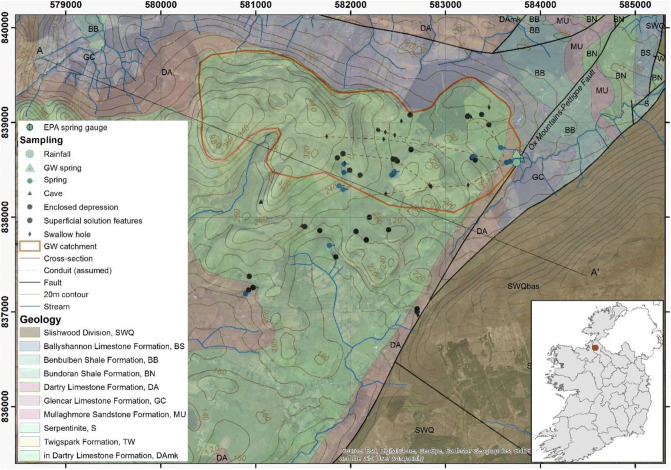
Geological map of the groundwater catchment of Manorhamilton, including karst features (source: Esri, DigitalGlobe, Earthstar Geographies, CNES and the GIS User Community)

The Manorhamilton spring catchment covers 3.6 km^2^ and ranges between 112 m above sea level (masl) at the spring and 392 masl at Mt Leean in the west. The topography of the catchment is shaped by rounded or hummocky grass-covered hills, with peat covering the upper part of the outcrop (MacDermot 1996). The catchment is highly karstified including many swallow holes. The entire catchment is underlain by the Dartry limestone formation which forms the aquifer discharging at Manorhamilton spring (Fig. 9). The catchment is bounded to the east by the Slishwood paragneiss and Ox Mountains-Pettigoe Fault where the Manorhamilton contact spring is located. Tracer tests confirmed a very shallow vadose zone and conduit transport velocities of 88–257 m/h. The mapped swallow holes indicate the presence of west–east aligned conduits.

#### Hydroclimatic sampling and data

High-frequency spring discharge data (15-min intervals) since April 2009 were available from the Environmental Protection Agency (EPA 2019). A tipping bucket ARG100 rain gauge (Environmental Measurement Ltd) was installed at the spring to record rainfall between December 2017 and June 2019. Between March and June 2018, the sampler (or logger) appeared to malfunction, not fully capturing all rainfall. Other climate data were obtained from the Met Éireann national meteorological station at Markree, located 19 km south-west of the spring. Maximum and minimum daily air temperatures were used to estimate the potential evapotranspiration (ET) following Hargreaves (1994). The average annual rainfall over the hydrological years 2010–2018 was 1,567 mm (1,023 mm effective rainfall) and average annual air temperature was 9.1 °C.

### Characterisation of aquifer responses

The ‘average’ heterogeneity of the aquifer with regard to recharge and flow was determined from the outlet of the aquifer, applying hydrograph decomposition and time series analysis on spring discharge time series. As a first step, a master recession curve (MRC) was established by overlapping and averaging several recession segments of a hydrograph, which were not impacted by recharge (rainfall). The MRC was then related to rainfall, infiltration into and storage within the aquifer (Mangin 1975; El-Hakim and Bakalowicz 2007). In order to represent the multiplicity of porosity expressed by (the drainage of) linear reservoirs, exponentials were fitted along the MRC following Maillet (1905). Three exponential components perfectly represent the overall MRC, which may suggest symmetry of the fracture network (Kovács and Perrochet 2008). Here, the lowest exponential is conceptualised to relate to diffuse groundwater recharge and flow (Geyer et al. 2008), with its recession constant *k*. This *k* value informed a two-parameter digital recursive filter (Eckhardt 2005; Rimmer and Hartmann 2014) in establishing a continuous baseflow signal. The ratio of baseflow to total discharge parameter (BFI_max_) was fitted so that the simulated baseflow matches exponential components; hence, neither *k* nor BFI_max_ can actually be considered as ‘free variable’ as usually considered.

### Modeling

#### Pipe network

**Fig. 10:**
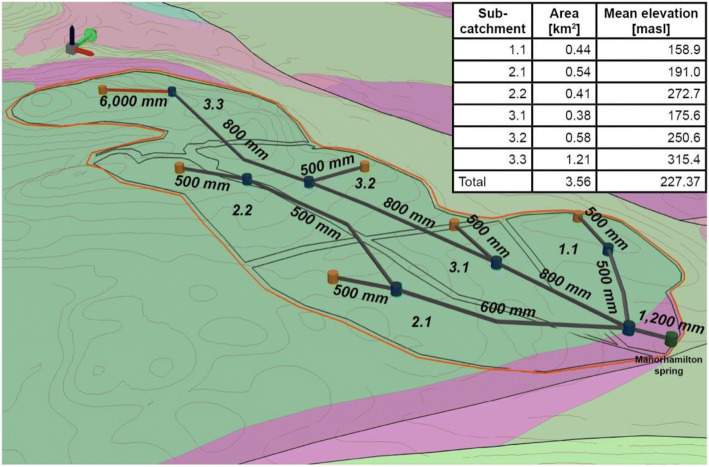
Schematic of the karst pipe network model, superimposed on the catchment geology and topography, consisting of six subcatchments (1.1–3.3) contributing recharge components to a networks of permeable (red) or full (dark grey) pipes connected by manholes (blue cylinders) finally discharging at Manorhamilton spring. The pipe diameters (mm) are displayed along the network

The general approach to designing the architecture of the semidistributed pipe network model built using the InfoWorks ICM software follows the description of section ‘Semidistributed pipe network models (development in Ireland)’. The morphometry of the network was based upon the alignments of the sinkholes (Fig. 10). The aim of this model was to represent the heterogeneity of recharge and associated flows, following the calibration procedure outlined as follows: (1) matching the overall water balance; (2) matching the recessions of the fast, intermediate and slow recharge component against the recession constants *k* obtained from the MRC; and (3) matching the simulated discharge against the observed discharge. The warm-up, calibration and validation periods were 15–31 December 2017, 01 January to 31 December 2018, and 01 January to 04 June 2019, respectively. The fast, intermediate and slow recharge components were taken from the ‘ICM runoff”, ICM soil store’ and ‘ICM Groundwater store’ (see Fig. 3) respectively for comparison against the recession constants *k* obtained from the MRC.

#### MODFLOW-USG with CLN

MODFLOW Unstructured Grid (USG) has been developed (Panday et al. 2013) to allow a better flexibility in grid design, enabling different grids, structured or unstructured, nested grids, and a variety of cell shapes (triangles, rectangles, hexagons for example) to be incorporated within the same model. These flexible grids can have an increased resolution along hydrogeological features (around rivers, wells, karst conduits etc.). The model used includes a CLN process which enables 1D connected features to be incorporated, in this case simulating flow within the karst conduits, such that the equations are solved simultaneously with the more general groundwater flow equations (Kresic and Panday 2017). These features have allowed a full distributed model of a karst catchment to be undertaken, as investigated in this study. Within the cylindrical conduits, flow can be simulated either as laminar (using the Hagen-Poiseuille equation) or turbulent (with optional formulations: Darcy-Weisbach equation, Hazen-Williams equation or Manning’s equation). The flow between the conduits and the surrounding aquifer cells is constrained through different options—effective leakance value, skin conductance and thickness, or use of a Thiem solution.

The formulation selected for the flow conditions within the conduits (CLN) was Manning’s equation (turbulent flow). For flow between CLN cells and connected groundwater flow (GWF) cells, it is possible to solve one nonlinear equation for each GWF connection. Here the flow between the two domains was computed from skin conductance and thickness for all CLN polylines. The interaction flow *Γ*_cpn_ between conduits and the surrounding aquifer (between CLN cells and GWF cells) is expressed by the following equation (Panday et al. 2013):

1 $${\varGamma}_{\mathrm{cpn}}={\alpha}_{\mathrm{cpn}}{f}_{\mathrm{upn}}\left({h}_{\mathrm{p}}-{h}_{\mathrm{n}}\right)$$

where*α*_cpn_is the saturated conductance between the CLN cell n and the GWF cell p,*h*_n_ is the head in the CLN cell,*h*_p_ is the head in the GWF cell, and*f*_upn_ is the wetted fraction of the upstream perimeter function of the flow depth.

Equations presenting alternative options, different available geometries, and convertible cells can be found in the aforementioned reference (Panday et al. 2013). In this study the conduits were chosen as cylindrical angled conduits. The Sparse Matrix Solver (SMS) Package was used to solve the sets of equations (nonlinear solution method).

**Fig. 11:**
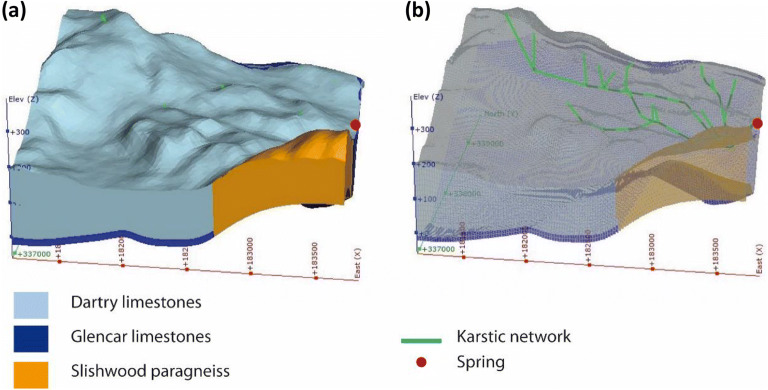
**a** 3D geological structure of the MODFLOW USG-CLN model at Manorhamilton with **b** locations of embedded karst network and spring

A conceptual model was first developed from the fieldwork investigations of the catchment and time series analysis of the spring discharge data. This defined the main infiltration points, direct connections proven by tracer tests, and characterisation of the different flow regimes. The architecture of the conduit network was very similar initially to that used in the semidistributed pipe model (section ‘Pipe network’) guided by the alignment off the swallow holes. A geological model was built using the software MOVE (Petex) using all available data (geological maps, field surveys, borehole logs, geological dip data, available cross-sections in the literature). This geological model was then exported to the software Leapfrog Geo (Seequent) which includes a hydrogeological module that creates a grid for the geological model that is compatible with MODFLOW. This also assigns some hydrological properties of each formation (hydraulic conductivity, specific storage, specific yield, initial hydraulic head). This hydrogeological grid was then exported into MODFLOW-USG with CLN using Groundwater Vistas 7 (Environmental Solutions Inc.) as the graphic user interface, to carry out flow simulations (see Fig. 11).

Results of the flow simulations are analysed and visualised: (1) directly in Groundwater Vistas using mass balance tools and hydrographs, (2) in Leapfrog in order to better visualise the relationships between the potentiometric surface with the geological layers and the surface in 3D over time and (3) in R where different scripts were developed to analyse the discharge time series performance during calibration and validation. The full process was carried out several times, incrementally, starting from a simplified model (rough geology, simplification of the flow paths), towards a more refined, complex model. This updating process also helped to refine the conceptual model of the catchment. The initial values for the different parameters were estimated from field data (including tracer tests) or available literature data (from previous pumping tests in the same geological formation for example). The final hydraulic conductivity used for the cells was 0.4 m/day.

## Results and discussion

### Model performances

**Fig. 12:**
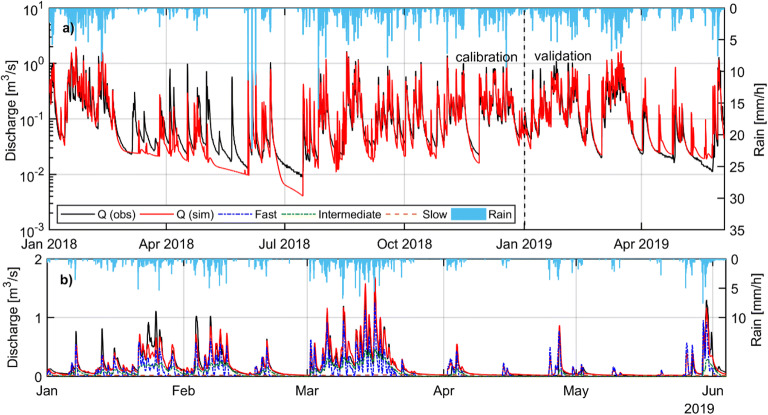
**a** Observed and modelled discharge of Manorhamilton spring and observed hourly rainfall during calibration and validation; **b** Observed and modelled spring discharge during validation as a result of the pipe flow dynamics as well as the three recharge components, namely fast (‘ICM runoff’), intermediate (‘ICM soil store’) and slow (‘ICM groundwater store’)

**Fig. 13:**
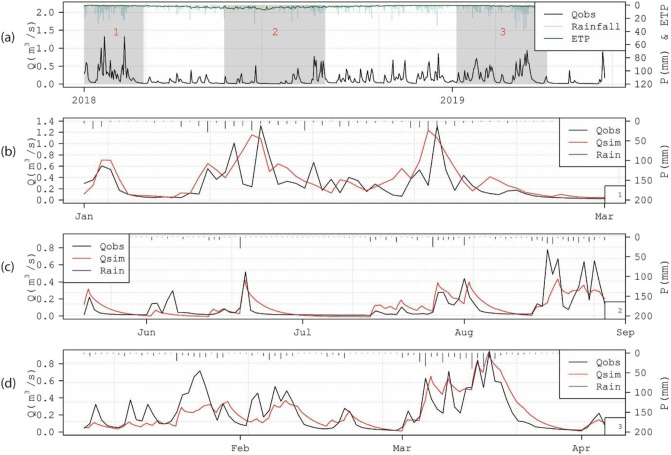
**a** Manorhamilton spring outflow January 2018 to May 2019 with the three periods shaded in grey which were those simulated by the distributed MODFLOW-USG-CLN model with results shown for **b** January to March 2018, **c** May to September 2018 and **d** January to April 2019

The simulated spring results from the models were compared to the spring discharge data across 2018/2019: for the full 18 months for the semidistributed model (see Fig. 12) and for three periods shaded in grey, totalling 9 months, for the distributed MODFLOW model (see Fig. 13). It was not possible to run the distributed model for the full 18-month period due to the model not converging for longer time series using the beta version of MODFLOW-USG with CLN software that was being used. The evaluation of the models’ spring flow simulations against the flow time series data was carried out using different performance indicators. For overall performance estimation, the following metrics were selected: the Nash-Sutcliffe efficiency (NSE; Nash and Sutcliffe 1970), the Kling-Gupta efficiency (KGE) (Gupta et al. 2009), and the volume conservation criteria (VCC). To assess low flow performance, the log-transformed NSE and Root Mean Squared Logarithmic Error (RMSLE) were calculated (De Vos and Rientjes 2007).

#### Semidistributed pipe network model

Figure 12a illustrates the modelling performance of the total spring discharge during calibration and validation. During calibration, the NSE and KGE are 0.807 and 0.904. The simulated results differ, however, from the observed time series again between March and the start of June 2018 which, as previously mentioned, is believed to be the result of erroneous rainfall recordings. During validation, the NSE increases to 0.882 while the KGE slightly drops to 0.892. Notably, the simulated discharge exceeds the observed discharge by 9.04% during validation, which again is most likely the result of the underrepresented rainfall during calibration. Figure 12b adds detail by displaying the three recharge and flow components that generate the final spring discharge signal. The recession of the fast and intermediate components could be matched well against the MRC with *k* = 0.03 h^−1^ and *k* = 0.15 h^−1^, respectively. Further, the simulated slow and conceptually diffuse component could reasonably be matched against the ‘observed’ signal represented by the baseflow component established by a digital recursive filter.

#### Distributed MODFLOW-USG-CLN model

Figure 13 shows the modelling performance of the total spring discharge for the three simulation periods shaded in grey in Fig. 13a. The NSEs and KGEs are generally between 0.5–0.6 (Table 1) with the exception of a very low NSE for period 1 which is mainly attributed to a shift in the peak flows which the NSE parameter is much more sensitive to compared to the KGE metric. In general, it has proved much more challenging with this distributed modelling approach (compared to the semidistributed model) to match the resolution of the spring discharge peaks (which in reality seem much more responsive to rainfall events) and their recessions. Although the model is very accurately simulating the volume of flows coming through at the spring over the three simulated periods, it is exhibiting a more damped response to the rainfall than what was monitored in the field.

**Table 1:** Performance of the simulated discharge compared to the observed spring discharge of Manorhamilton spring for the semidistributed and distributed models

Performance metric	Semidistributed (InfoWorks) model		Distributed (MODFLOW USG-CLN) model		
	Calibration	Validation	Period 1	Period 2	Period 3
NSE	+0.807	+0.882	+0.160	+0.517	+0.540
NSE LN	+0.663	+0.825	+0.490	+0.650	+0.440
KGE	+0.904	+0.892	+0.50	+0.59	+0.53
RMSLE	+0.068	+0.056	+0.72	+0.70	+0.69
VCC	0.21%	9.04%	0.3%	−0.28%	0.007%

**Fig. 14:**
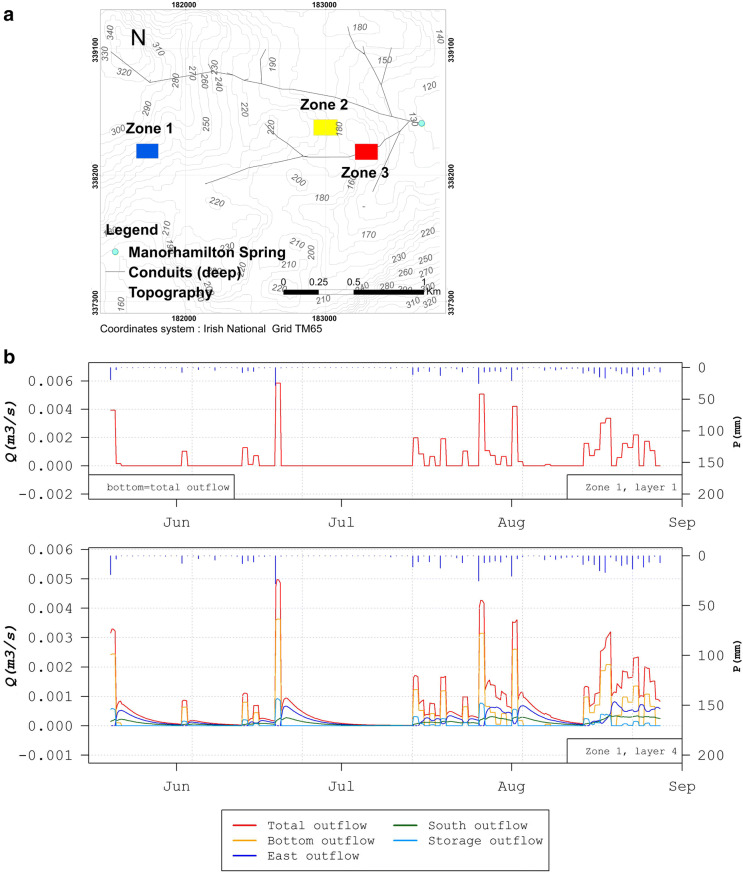

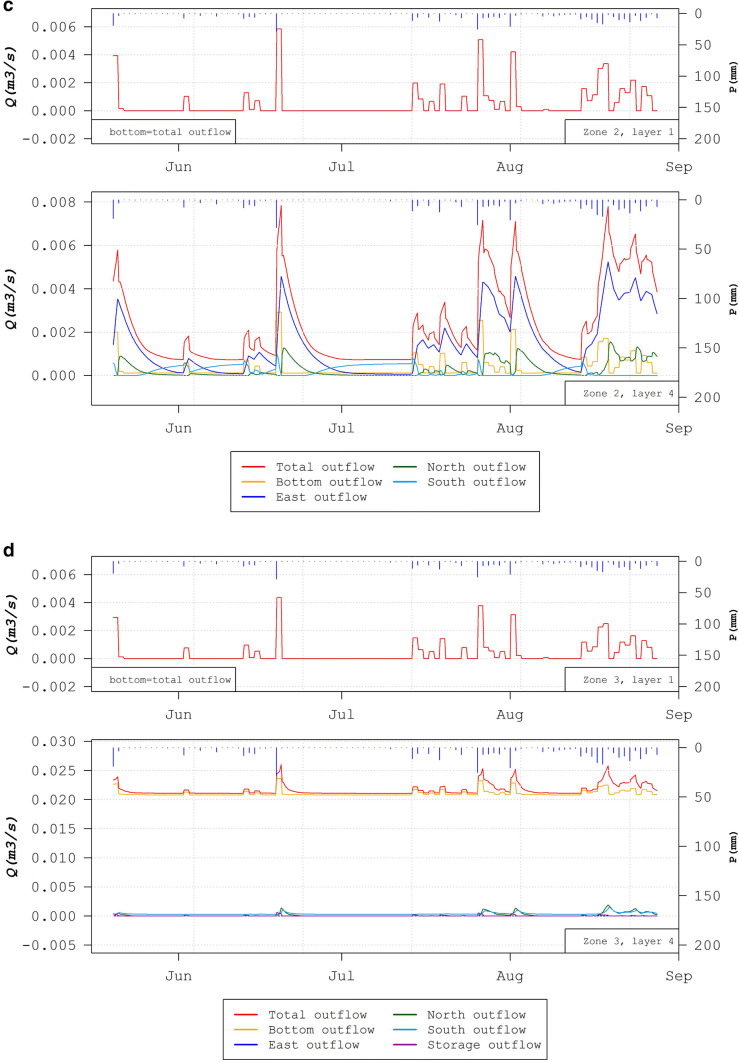
Diffuse flow at different outflow layers on the Manorhamilton catchment located as marked on the map (**a**). Time series of flow in three zones at different distances from the main conduit and flows between layers 1 and 4 are shown for **b** zone 1, **c** zone 2 and **d** zone 3

Finally, the ability of the model to show flows at any location within the aquifer is demonstrated. Results from the simulation of “diffuse” flux through aquifer cells at different distances away from the main line conduits are shown in Fig. 14. At all three locations (zones 1–3), the difference between the outflow from cells in layer 1 compared to layer 4 is shown, representing a depth difference of 94 m (zone 1), 40 m (zone 2), and 61 m (zone 3).

Zone 1 is located far away from any influence of any highly transmissive conduits and shows a similar flow profile with depth between layers 1 and 4, with just a slight lagged response from layer 4 (Fig. 14b). This area demonstrates the behaviour and contribution of the diffuse flow aquifer (outside of conduits)—small flow volumes with a “smooth” damped response to rainfall recharge, with water moving laterally as well as downwards. Zone 2 is located between two main line conduits, each 3.2 m diameter. Figure 14c shows higher net outflows coming out of layer 4 as the matrix is beginning to “feel” the influence of the pressurised conduits at higher flows, caused by the higher drainage (more dense potentiometric gradients) in the vicinity of conduits. The response is also much sharper (peaks) with more of a “karst signature”. Zone 3 is located above the main 2.4-m diameter conduit but also intersects a smaller conduit from a sinkhole at the surface of 0.6 m diameter, which connects to the main conduit below. The main conduit is just 8 m below layer 4 of zone 3 volume. The fluxes for zone 3 are much higher compared to zones 1 and 2 because the flows are concentrated towards this conduit draining from the surface to the main network first (the segment of that “feeding” conduit in layer 4). It is also observed that, in comparison with zone 2, here the majority of the diffuse flow is being drained vertically downwards (i.e. bottom outflow) towards the main conduit located 8 m below, whilst the lateral flows are negligible in comparison. Figure S4 of the ESM also shows the variations of the 3D potentiometric surface of the Manorhamilton karst catchment for the first simulation period (January 2018 to May 2019).

### Discussion

The two different modelling approaches (semidistributed versus distributed) applied on the same small karst catchment in Ireland have provided some interesting comparisons. The first is the difference between the resulting conduit networks for the two models that were required for optimum calibrations against the spring discharge time series. The morphometry of the two networks in the models is very similar as they are based on the alignments of sinkholes, but there are a few differences: the main one being the addition of two small branches of conduits close to the spring (north and south) in the distributed model. These modifications were added to lower the potentiometric surface near the spring to prevent the surface flooding that was being simulated, which does not happen in reality. This is also consistent with field observations as there is a small nonperennial spring in that area suggesting the presence of conduits near the surface and denser network. A more striking comparison, however, is the significant difference in size of the main conduits between the two models. The semidistributed model consists of three main tributaries (ending in 0.5, 0.6 and 0.8-m diameter conduits all feeding into a final conduit of 1.2 m diameter before the spring (Fig. 10), whereas the conduits in the distributed model are approximately twice the size ranging from 1.0 up to 3.6 m diameters along the three main tributaries. The balance between the hydraulic conductivity of the matrix cells and the density and diameter of the conduits is obviously critical for such a distributed modelling approach and needs more investigation in the future to try to achieve better matches to the spring outflow.

Some more automated approaches have been developed to characterise the 3D conceptual models of karst aquifers, such as KARSYS conceptual model approach (Malard et al. 2015), which could help to define the conduit network morphometry before being incorporated into these distributed (or semidistributed) models. Groundwater Vistas 7 (GW7) is also currently being modified to allow CLN as targets to calibrate automatically against spring discharge. This had to be done manually for this research, as well as externally from the software which did not allow plotting the spring discharge in the beta version of the software used, which made the whole process very time consuming.

Another possible way to strengthen and improve the performance of the distributed model could be achieved by testing different formulations, for example using the Preissmann slot concept to incorporate pressurised flow. This might enable one to better reproduce the transition between free surface flow and pressurized flow, like has been done in different studies using the US Environmental Protection Agency Storm Water Management Model (SWMM) for urban drainage networks (Ferreri et al. 2010) but also in karst environments (Gabrovšek and Peric 2006; Halihan et al. 1998; Perne et al. 2012; Peterson and Wicks 2006) as well as the InfoWorks ICM software used in this paper (see section ‘1D models to simulate groundwater/surface-water interaction’) . In the current version of MODFLOW-USG CLN, the alternative is to use confined flow within CLN to account for saturated conditions (but between CLN only), and through the use of convertible cells to simulate “flow to dry cells” between CLN and GWF cell. This enables flow exchange between CLN and GWF cells in both directions and wet and dry cells; however, there is not an explicit pressurised formulation available. Assessing the difference that could result in the flow path and dynamics could be a perspective of this work.

Another possible bias is the definition of the conduit network geometry: it has been defined based on surface fieldwork observations, analysis of the 3D geology and comparison with similar systems, but there are some uncertainties regarding tortuosity and depths. Applying a more extensive study on the possible variations of the network using, for example, a generator based on a fracture network, like the Stochastic Simulator (SKS; Borghi et al. 2016, 2012; Sivelle et al. 2020), might help to refine optimal configurations for the karst network. Finally, the calibration process has not yet been automated in this case and investigating multi-parameter calibration could also yield new possibilities.

Overall, the semidistributed model was undoubtedly quicker to set up and calibrate to achieve better performance statistics at the spring; however, some of this difference in time and effort required could be attributed to the fact that a beta version of MODFLOW-USG with CLN was being used, and this modelling exercise on such a karst application generated a lot of useful discussion and feedback for the software developers. Finally, the ability of the distributed model to simulate diffuse flows across any parts of the aquifer was demonstrated which, as highlighted earlier, has been a limitation of the semidistributed modelling approach. This has shown the influence that the conduits can have on more diffuse recharge areas in their proximity. This also presents the potential to model different types of contaminant inputs across the catchment and assess their transport and attenuation characteristics which should be of significant interest for water management and policy makers. The simulation of different contaminant plumes (solutes, microorganisms etc.) at different points across the catchment is a focus of ongoing work using this model.

## Conclusion

This paper has detailed the development of a semidistributed modelling approach for karst aquifers using urban drainage software, particularly in an Irish context. The models have proven to be very useful for different applications with examples given for the ecohydrology of ephemeral karst lakes, extreme groundwater flood alleviation, karst network investigations, submarine groundwater discharge and quantification of different recharge and flow components. The limitations of the approach are also highlighted, in particular not being able to simulate explicitly more-diffuse infiltration and flow paths (and linked contamination) on an areal basis as well as the missing storage component that can receive inflow at times of pressurized conditions of the conduits. Hence, a more distributed, finite-difference modelling approach using MODFLOW Unstructured Grid (USG) with the newly developed CLN process has been compared against the semidistributed approach on the same karst catchment. Whilst it has proven difficult to achieve the same levels of model performance in simulating the spring flows in the distributed model compared to the semidistributed model, the ability to interrogate the flow paths at any point on the 3D aquifer is demonstrated, which has given new insights into the influence of highly transmissive conduits on the surrounding matrix in such complex systems.

The choice of modelling approach taken usually depends on the main objective for which it is being developed: to simulate spring flow (from a water resources perspective), to evaluate the ecohydrology of wetlands, to simulate and predict groundwater/surface-water interaction (i.e. flooding), to evaluate water contaminant transport and attenuation processes, etc. The semidistributed approach has proved very useful in a range of applications and is relatively quick to build the models and test different hypotheses in a conduit-driven karst aquifer scenario. However, it does have limitations if trying to model diffuse contamination on a catchment from a water quality perspective, which could be achieved using a more distributed modelling approach. One conclusion from this work is that a two-step process—such as the semidistributed system to define the main conduit pipe network (matching the spring hydrographs), which can then be taken forward for incorporation into a more distributed model structure if further levels of interrogation are required across the catchment of the aquifer from a water management perspective—might provide an optimal approach for a new karst system.

## Electronic supplementary material

ESM 1 (PDF 1519 kb)
