# Supplementary material for: An evaluation of semidistributed-pipe-network and distributed-finite-difference models to simulate karst systems
Source: Hydrogeol J. 2020 Nov 11;29(1):259–79. doi: 10.1007/s10040-020-02241-8 (PMC7870641; doi:10.1007/s10040-020-02241-8)
Supplement: Supplementary file 1 — (PDF 1519 kb) [file 10040_2020_2241_MOESM1_ESM.pdf]

Hydrogeology Journal – Electronic Supplementary Material

An evaluation of semidistributed-pipe-network and distributed-finite-difference models to simulate karst systems

Gill L.W. [1]*, Schuler, P.[1]*, Duran, L.[1], Morrissey P.[1], Johnston P.M.[1]

[1] Department of Civil, Structural and Environmental Engineering, University of Dublin Trinity College, Dublin 2, Ireland

*corresponding Author: Laurence Gill

Email: laurence.gill@tcd.ie, Telephone: +353(0)18961047

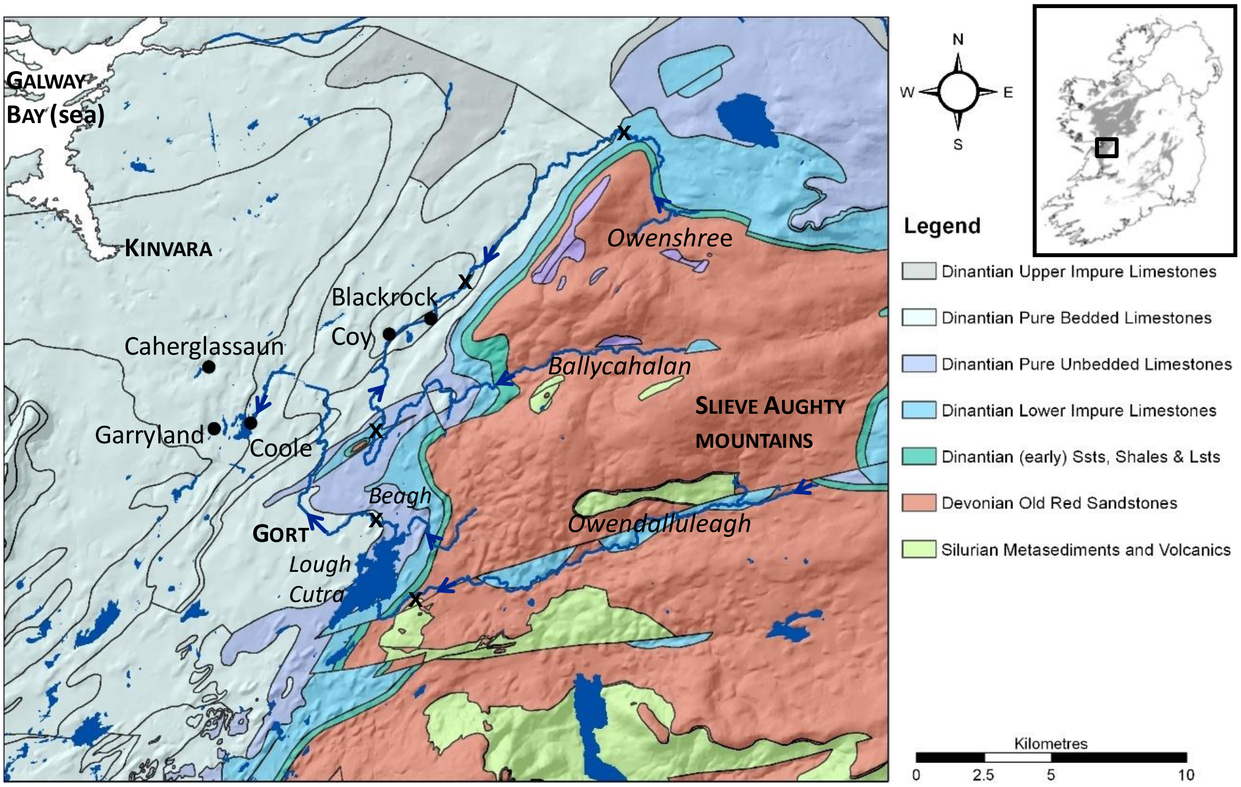

Figure S1. Geology and relief map of study area showing five turloughs (•), the main rivers draining the Slieve Aughty mountains (gauging stations marked **X**) and the main spring at Kinvara (inset shows limestone geology of Ireland and location of study area) (Gill et al., 2013).

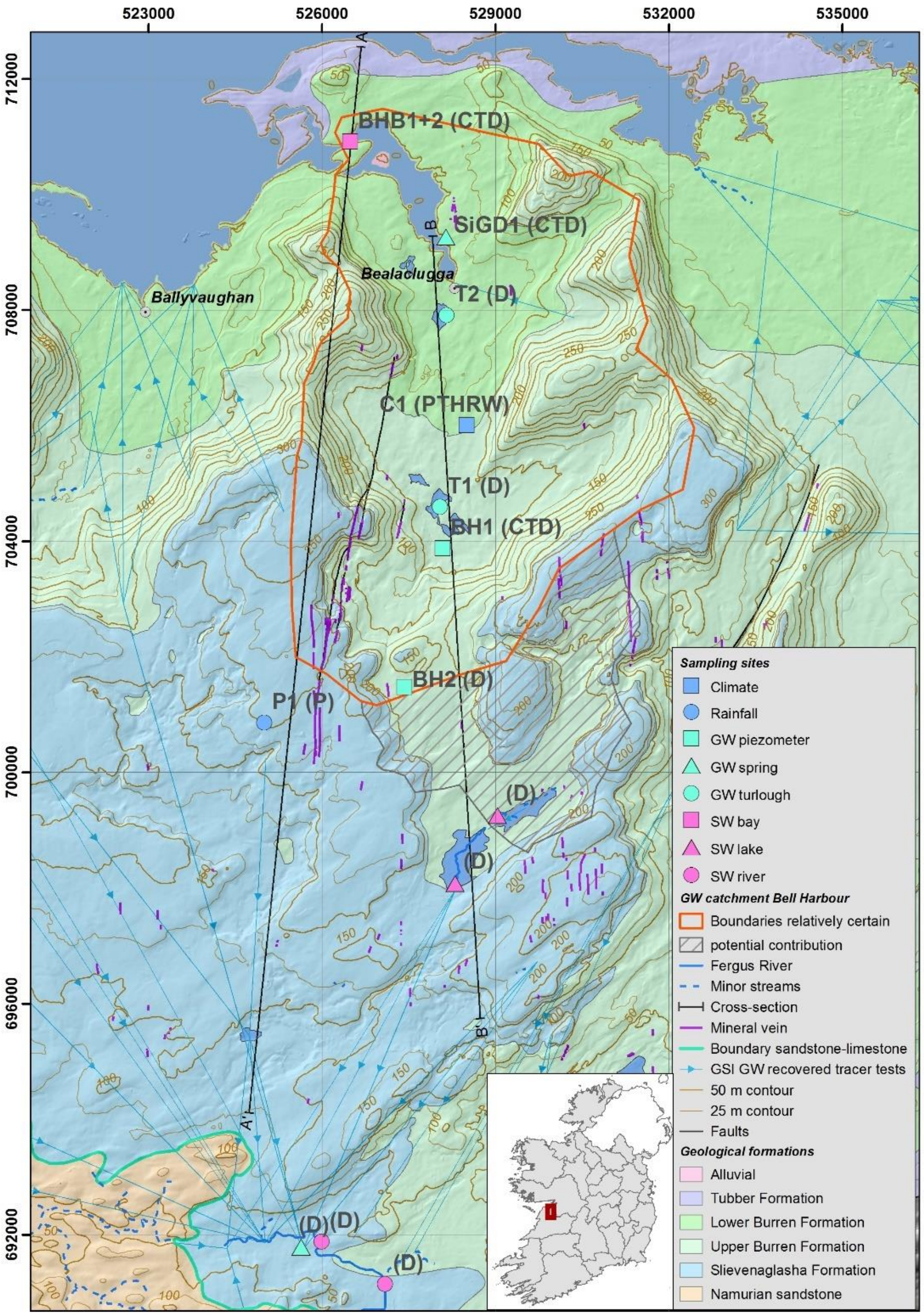

Figure S2. Bell Harbour showing minimum groundwater catchment and potential additional contribution in the south in the Burren limestone plateau: topography, geology, structure, tracer tests, sampling locations (C = conductivity, D = depth, T = temperature, P = precipitation, H = relative humidity, R = net radiation, W = wind speed/direction). (Schuler et al., 2018).

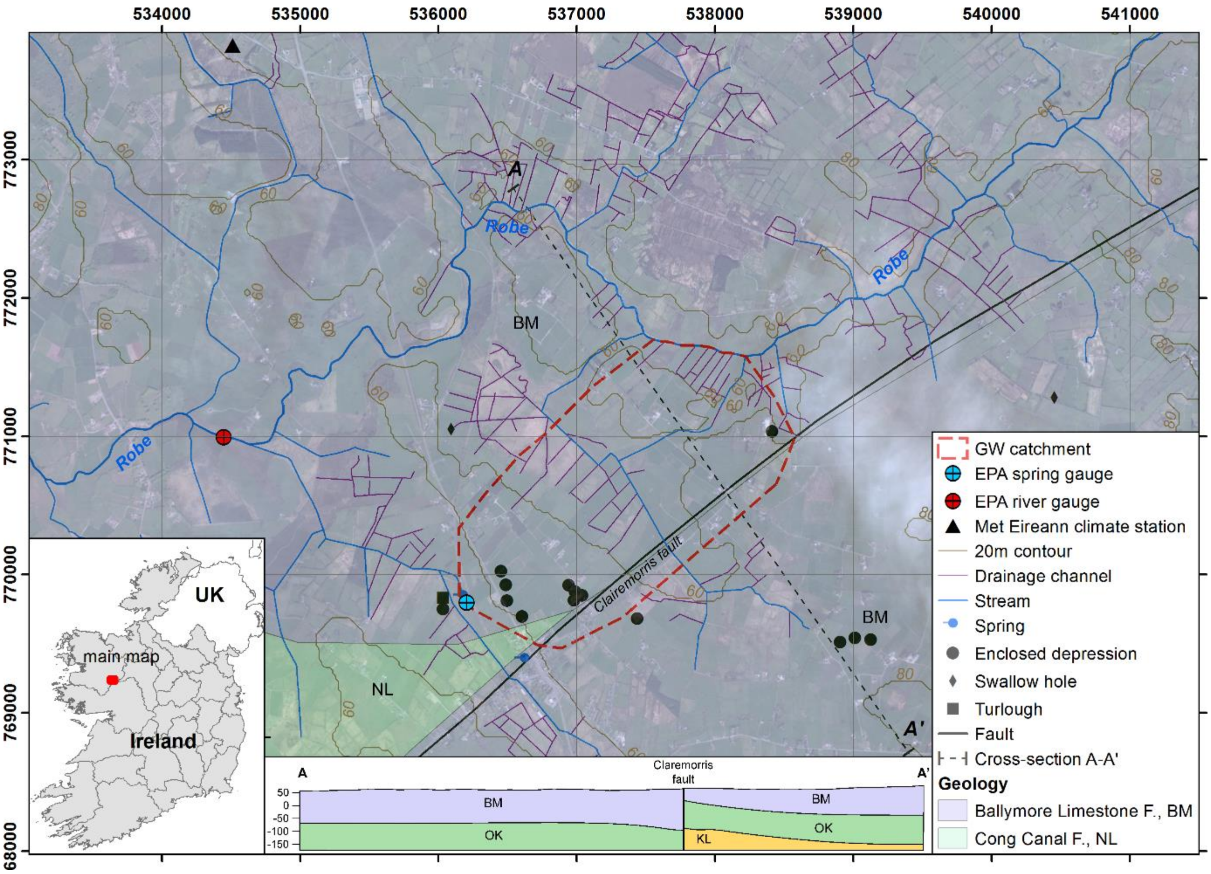

Figure S3. Geology of the Ballindine karst aquifer catchment (Schuler et al 2020).

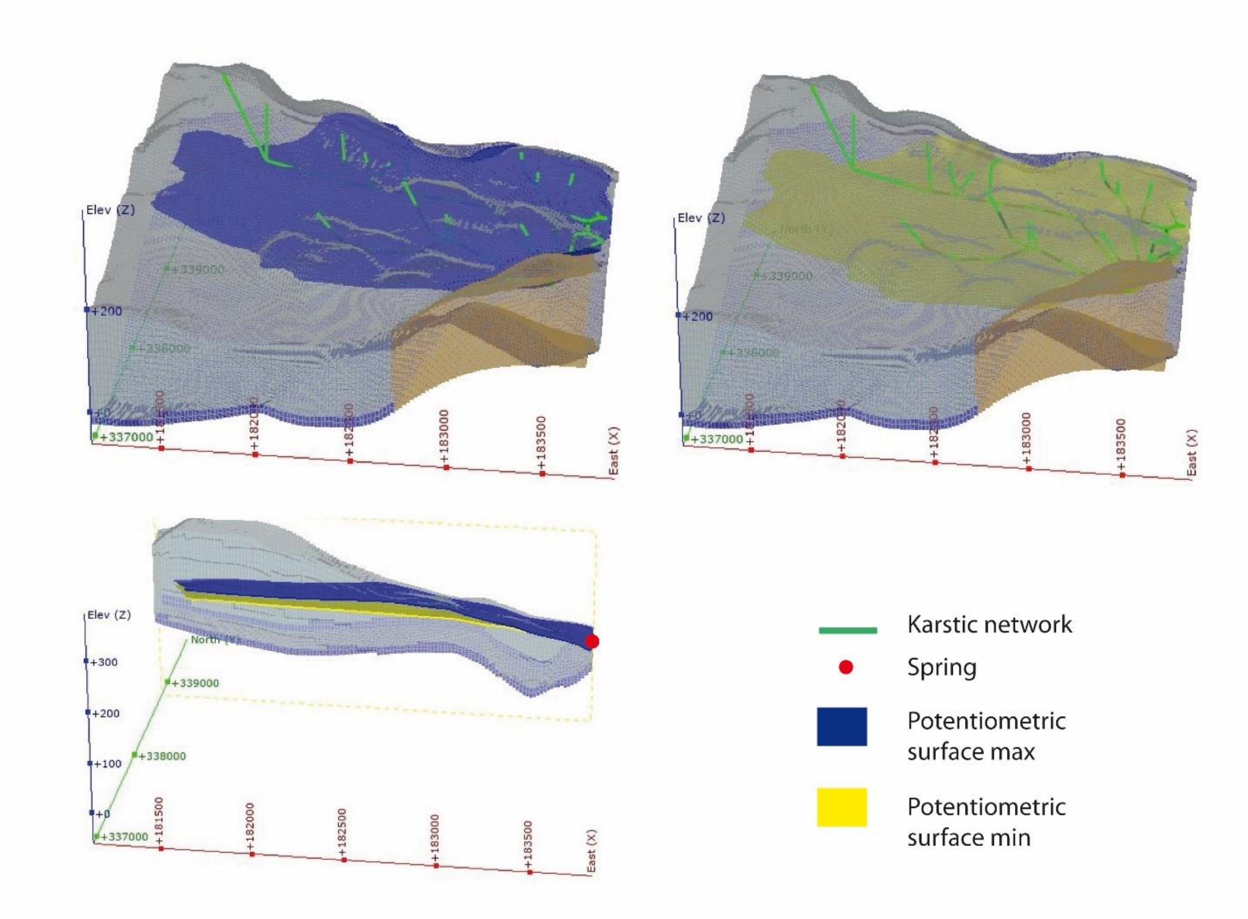

Figure S4. Variations of the potentiometric surface of Manorhamilton spring outflow for the first simulation period (January 2018 to May 2019) by the distributed MODFLOW USG-CLN model.

ESM References

Gill LW, Naughton O, Johnston PM (2013) Modeling a network of turloughs in lowland karst. Water Resources Research 49(6), 3487-3503. https://doi.org/doi:10.1029/wrcr.20299

Schuler P, Duran L, McCormack T, Gill L (2018) Submarine and intertidal groundwater discharge through a complex multi-level karst conduit aquifer. Hydrogeology Journal 28: 2629-2647 http://dx.doi.org/10.1007/s10040-018-1821-3

Schuler P., Cantoni E., Duran L., Johnston P., Gill L.W. (2020). Using wavelet coherence to characterize surface water infiltration into a low-lying karst aquifer. Groundwater 58(4) http://dx.doi.org/10.1111/gwat.13012
